# Acetone sensing in liquid and gas phases using cyclic voltammetry

**DOI:** 10.1038/s41598-022-15135-4

**Published:** 2022-06-30

**Authors:** Yusra Obeidat, Abdel Monem Rawashdeh, Ayman Hammoudeh, Rawan Al-Assi, Ahmad Dagamseh, Qasem Qananwah

**Affiliations:** 1 Electronic Engineering Department, Hijjawi Faculty for Engineering Technology, Yarmouk University, Irbid, 21163 Jordan; 2 Department of Chemistry, Faculty of Sciences, Yarmouk University, P.O. Box 566, Irbid, Jordan; 3 Department of Biomedical Systems and Informatics Engineering, Hijjawi Faculty for Engineering Technology, Yarmouk University, Irbid, 21163 Jordan

**Keywords:** Biochemistry, Biological techniques

## Abstract

This paper presents the use of cyclic voltammetry to measure acetone concentration in liquid and vapor forms at disposable screen-printed electrodes of platinum working electrode, platinum counter electrode, and silver/silver chloride reference electrode. The main characteristics of the acetone sensor including its linearity, sensitivity, reproducibility, and limit of detection (LOD) were studied by doing different experiments to test both liquid and vapor samples in the physiological range of 1 µM to 10 mM. The change in acetone concentration was monitored by comparing the lineshape of butterfly region before and after injecting the acetone sample in the baseline solution that contains 0.5 M H_2_SO_4_. The sensor was shown to have a good sensitivity, reproducibility, and a linear response with respect to the acetone concentration in both liquid and gas phases over a range of 1 µM to 10 mM with R^2^ > 0.97 and LOD of 0.1 µM. The system stability was improved by building a closed glass system to reduce the exchange of acetone with the surrounding air in an open environment. The closed system was tested using vapor samples and the error bars in the calibration curve were reduced to more than half of their values before using the closed system. The new system will be used extensively in future for an enzyme-based acetone sensor that will be used for diabetes monitoring.

## Introduction

Acetone ((CH_3_)_2_CO) is one of the building blocks that are common in organic chemistry and widely used in industries and labs such as in cleaning, purifying paraffin, methacrylate’s synthesizing, dissolving plastic, in various pharmaceutical and cosmetic products, and tissues dehydrating^1–3^, however, Acetone inhalation is harmful to the health especially to the nerve system and has other effects including narcosis, headache, and fatigue^4–6^. Moreover, Acetone is a fat metabolic nature that acts as a by‐product of mammalian metabolism^7–9^, and the concentration of acetone in obese people is higher than that in the slim people.

The level of the breath acetone can increase due to many factors such as: the subject’s cardiorespiratory fitness during and after exercising^10^, diabetes mellitus as it can reach more than 1800 ppm in diabetics, extensive physical activity^11^, and it can reach hundreds of ppm during ketogenic diets^2,12^. The increase in the exhaled acetone is also associated with poor prognosis and has been described as a biomarker for heart failure. Therefore, it is important to measure the breath acetone and correlate it to different types of diseases to be able to diagnose the patient’s health conditions.

The acetone gas sensors that are commercially available work only in the range of 50–5000 ppm which is much higher than the physiological range^13^, thus we need to develop some techniques to measure the breath acetone in this range. Most of the common techniques that are used for measuring the breath acetone concentration in the physiological range were extensively reviewed by Obeidat et al.^14^. Some of these techniques are based on spectrometry and known of their high sensitivity and reliability, such as Gas chromatography-mass spectrometry (GC–MS)^15–17^, ion mobility spectrometry-mass spectrometry (IMS-MS)^18,19^, Proton Transfer Reaction Mass Spectrometry (PTR-MS)^20,21^, Selected Ion Flow Tube-Mass Spectrometry (SIFT-MS)^22–24^, cavity ringdown spectrometry^25–27^, and laser photoacoustic spectrometry^28–30^, but these techniques are expensive, not portable, and not suitable for real time daily monitoring. Other techniques such as resistive gas sensors which are widely used in application of low-frequency (flicker) noise^31–33^, optical-fiber sensors^34–36^, semiconductor sensors^37,38^, piezoelectric sensors^39–41^, luminescence-based sensors and chemiluminescence-based sensors but these sensors have low selectivity. Colorimetric acetone sensors have shown a great performance in real breath and can be read-out by naked eye^42,43^.

In recent years, the technology moves toward miniaturized sensors such as chemical sensors^44–49^, electronic noses^50–52^, and electrochemical gas sensors^53–62^ which make it attractive to be used in building portable and cheap devices for diabetes monitoring. The electrochemical sensors are known of their simplicity, cheap cost, and good selectivity, and they are very attractive in designing affordable portable devices with disposable sensors^1,2^. Therefore, they can be a good choice for building a handheld device for invasive real-time diabetes monitoring^1,2,7,54,59–62^.

The electrochemical sensor used for measuring acetone are mainly two types: enzymatic and non-enzymatic. The enzymatic acetone sensor requires an enzyme system that produces H_2_O_2_ when acetone vapor or liquid is added, this generated H_2_O_2_ can be detected using amperometry or voltammetry^2,54,55^ and the current measured in response to the reaction is proportional to the input acetone concentration. A palm-size electrochemical sensor was designed by Landini et al.^54,55^ to measure the concentration of acetone in the breath of dieting and exercising healthy individuals using Amperometry. The sensor has a reusable palm-size handset to insert a disposable mouthpiece that contains a fresh single-time use enzyme electrode sensor; this mouthpiece is for the user to exhale in. The range of acetone in human breath that was measured using this sensor is from 0.2 to 10 ppm^55,63^.

The non-enzymatic breath acetone sensors need specific electrolytes such as Sulfuric acid (H_2_SO_4_) or Sodium tartrate (Na_2_C_4_H_4_O_6_) to form a reaction with acetone and generate an electrochemically active product that can be measured in redox reaction through amperometry or cyclic voltammetry (CV) and generate a current proportional to the concentration of breath acetone^1,7^. A non-enzymatic acetone electrochemical sensor was developed by Wang et al.^1^ using a lead foil electrode in 0.1 M sodium tartrate solutions where cyclic voltammetry was used to determine the necessary activation voltage of a range from − 2.21 to − 2.32 V (versus Ag/AgCl). Chronoamperometry were applied to analyze the electrochemical behavior for different acetone concentrations and have shown that the Pb foil electrode has a fast response time and a good linear relationship between response current and acetone concentration in the range from 50 to 250 ppm. Another non-enzymatic sensor was developed by Motsegood et al.^7^ using cyclic voltammetry for measuring the concentration of acetone in vapor phase and in solution at platinum electrodes in 0.5 M H_2_SO_4_. They have shown the ability to measure breath acetone concentration in the range of 1 μM to 10 mM by observing electrolysis of adsorbed acetone in the voltametric butterfly region.

In this work, we are presenting a characterization of acetone sensor in vapor and liquid phases in the range from 1 μM to 10 mM using screen printed electrodes of platinum working electrode, platinum counter electrode, and silver/silver chloride reference electrode. We have used cyclic voltammetry and tested acetone in both vapor and liquid forms, and the difference in current between the reduction peak of acetone and the baseline in the butterfly region was used to generate the calibration curve. Moreover, we have built a closed glass system to improve the system stability and reduce the amount of error caused by the exchange between the acetone and air in the open environment.

## Materials and methods

### Sensor electrodes

**Figure 1:**
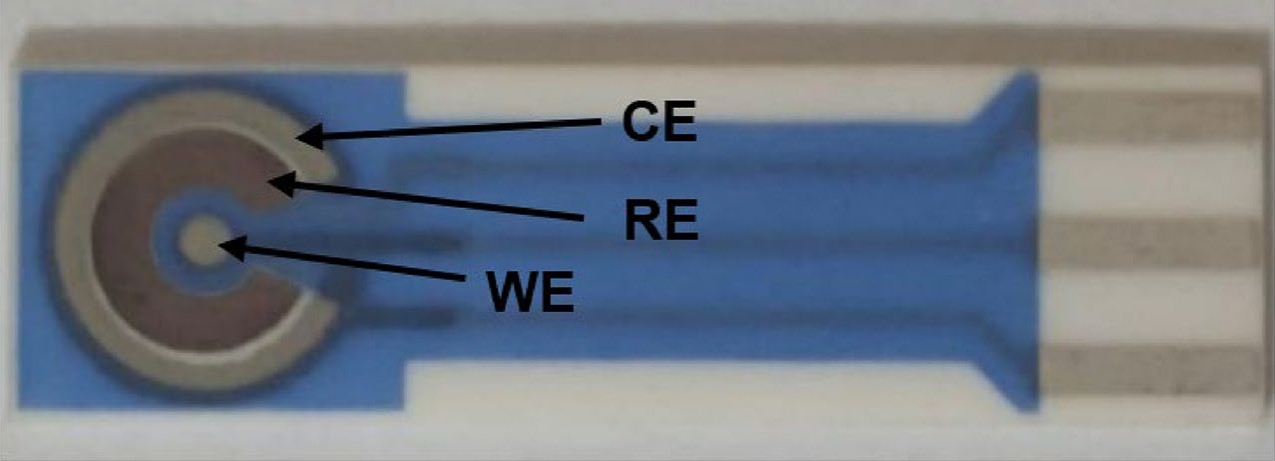
BVT screen printed electrodes.

All experiments used BVT screen printed electrodes (Palmsens, Netherlands). The electrodes were printed on corundum ceramic substrate of 25.4 mm length, 7.26 mm width, and 0.63 mm height. On this surface, a circular Platinum (Pt) working with diameter of 1 mm, a curved (Ag/AgCl) reference, and a curved counter electrodes are applied and at the end of the sensor there is a contacting field which is connected by silver conducting paths which are covered by a dielectric protection layer as shown in Fig. 1. The reason for choosing Pt working electrode is due to its high stability and good sensitivity to electrochemical reactions^64–67^.

### Reagents

Pure acetone and concentrated H_2_SO_4_ from (Sigma Aldrich, USA). The acetone samples were prepared in 250 mL flasks. The samples were injected using a 50 mL syringe. Deionized water from a Milli-Q plus water filtration system was used to dilute acetone solutions as well as to rinse the electrode surface between measurements.

### Electrochemical instrumentation

A single channel Gamry Potentiostat (International Enterprise Center, China) was used to perform cyclic voltammetry in all experiments. The Potentiostat was connected to a PC that has Echem Analyst software to display and save the CV data to generate the calibration curves.

### Electrode electrochemical cleaning

**Figure 2:**
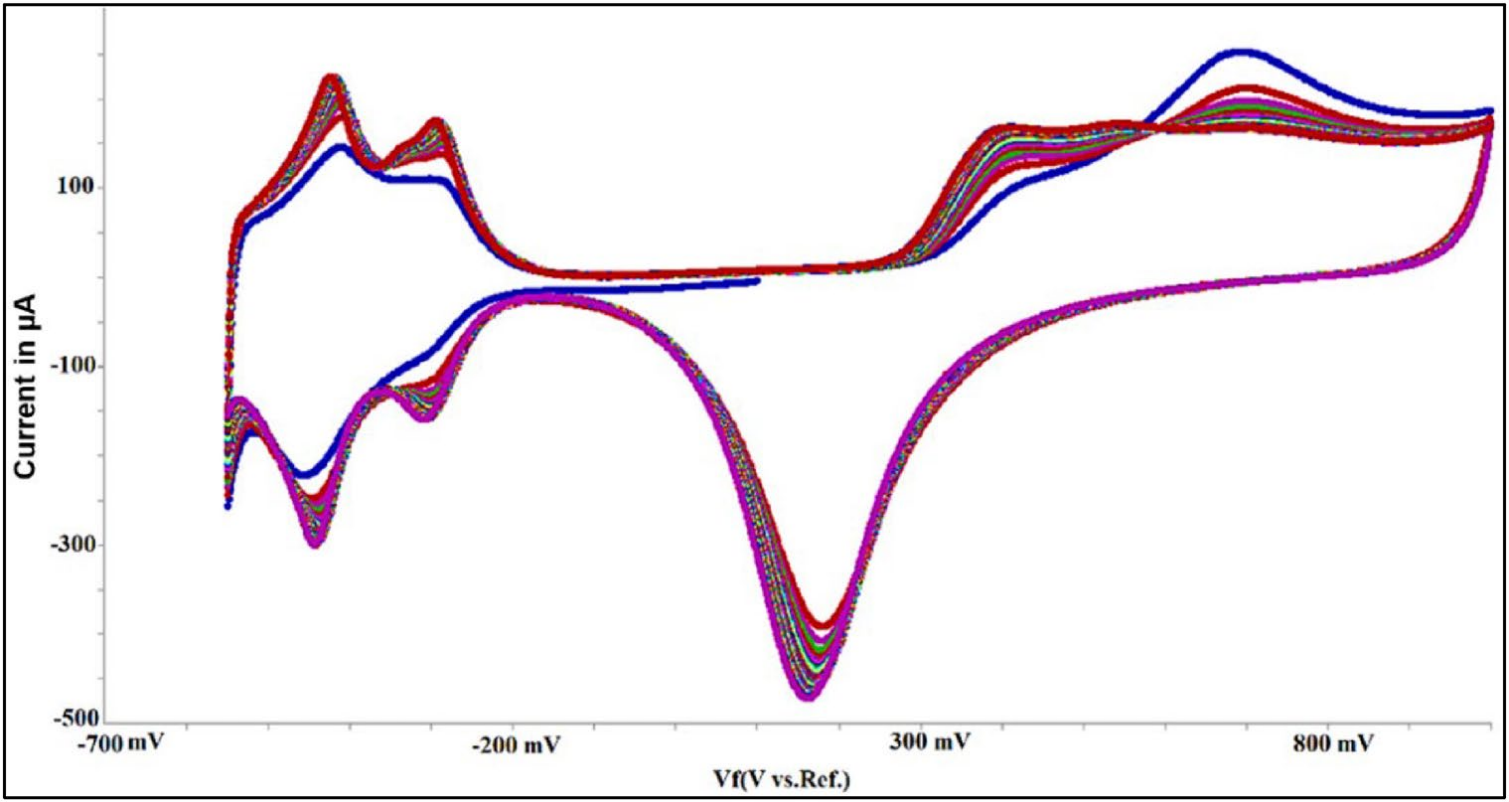
Cyclic voltammetry results at 500 mV/s and 50 cycles for cleaning.

Before each experiment, the electrodes were rinsed using DI water and dried using N_2_ gas, then electrochemically cleaned in 0.5 M H_2_SO_4_ by scanning between − 0.2 V and + 1.5 V at 500 mV/s for 50 cycles to generate the hydrogen reduction ‘butterfly’ wave on platinum electrode as shown in Fig. 2.

### Acetone sample procedure

Acetone samples were prepared in liquid and gas forms. The liquid samples were prepared in 0.5 M sulfuric acid using the common dilution process that is based on the equation *M*_1_*V*_1_ = *M*_2_*V*_2_^68^, where *M*_1_ is the *molarity*(*M*) of the stock acetone solution, *V*_1_ is the *volume* of acetone needed to be added from the stock solution, M_2_ is the concentration of the desired acetone sample, and V_2_ is the *volume* of the desired sample. We have made 1 L of 0.5 M sulfuric acid to be used as a blank solution (solvent) for dilution. We have prepared acetone stock of 13.5 M and then diluted it to obtain 50 mM acetone by mixing with H_2_SO_4_ solution in a 50 mL glass flask with a parafilm and septa used to close it to ensure stability. We have used the obtained 50 mM for making other concentrations in the range from 1 µM to 10 mM by dilution.

The gas samples were prepared at room temperature by mixing 25 mL acetone solutions of specific concentrations in a 50 mL glass flask with a parafilm and septa used to close it. The sample were left for 30 min to give it a time to generate the vapor sample and distribute equally in the air inside the flask to represent breath samples. Since the acetone vapor on top of the headspace directly depends on the solution concentration, all concentrations of the vapor phase samples used in the calibration process are reported as the acetone solution concentration in water.

### Closed system for stability improvement

**Figure 3:**
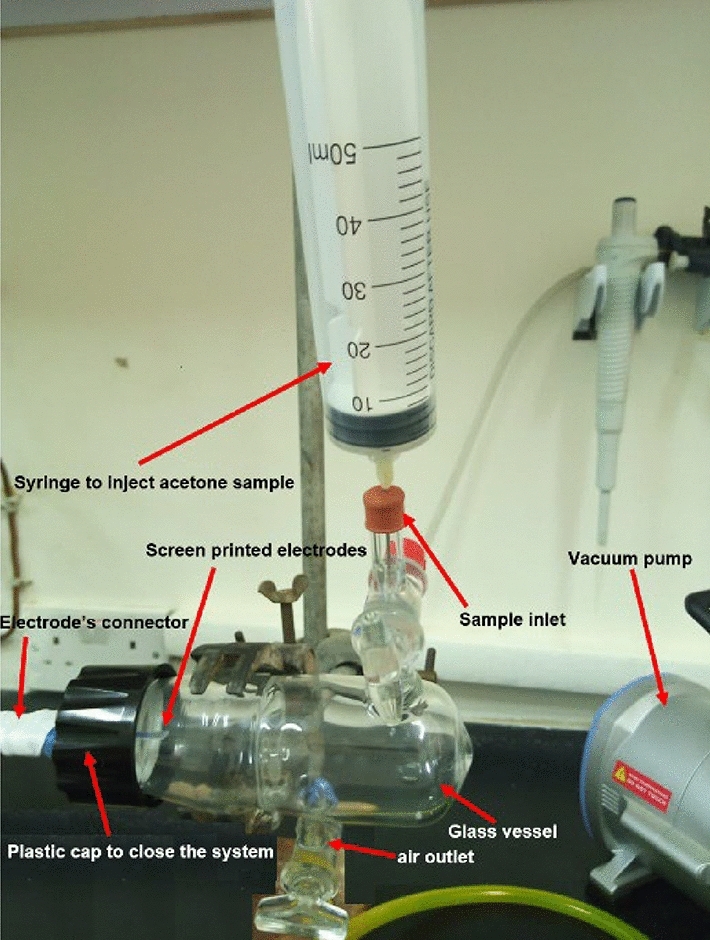
A complete setup for the closed glass system.

A closed glass system (Fig. 3) was made in the Chemistry lab (Chemistry Department, Yarmouk University) to improve the stability of the measurements. A Glass vessel has one side to put the electrodes in, air outlet to vacuum out any gas before the measurements, and sample inlet to inject the acetone sample. This system helps to reduce the exchange of acetone with air during measurements which ensure better stability and less errors.

### The electrochemical process

The electrochemical process used in this work for measuring the acetone concentration is based on the adsorption and electrochemical reduction of Acetone in Sulfuric acid (H_2_SO_4_) medium on Pt electrodes^7,69,70^. This process has been demonstrated in detail using on-line mass spectrometry^70^. As a result of this process, Acetone may be reduced to propane and isopropanol with a rate of formation depends on the applied voltage and on the electrode surface^69^.

Using 0.5 M H_2_SO_4_ is necessary to create a competitive adsorption between acetone and free protons^7^ that can be examined using cyclic voltammetry. The potential sweeping results in desorbing part of the adsorbed acetone molecularly while the other part hydrogenates to propane. The concentration of acetone is correlated to the effect of the adsorption of acetone in a 0.5 M H_2_SO_4_ solution on the line shape of the butterfly region. During this process, adsorbed acetone is reduced or desorbs from the surface and the lineshape is altered by blocking adsorption sites normally available to hydrogen, as a result a signature hydrogen reduction wave is regenerated^7^.

### Measurement setup

The experiments were done in OLED (Organic Light Emitting Diodes) lab (Chemistry Department, Yarmouk University).

The liquid and vapor samples were prepared using the protocols explained in “Acetone sample procedure”. After cleaning the electrodes using the protocol in “Electrode electrochemical cleaning”, we rinse the electrodes with DI water then dry with N_2_ gas. A baseline measurement is generated by injecting 25 µL of 0.5 M H_2_SO_4_ on top of the electrodes and scanning between − 0.2 V and + 1.5 V at 100 mV/s for 3 cycles. Then the electrodes are rinsed with DI water and dried with nitrogen.

In case of the liquid samples, each sample (1 µM to 10 mM) was tested by injecting 25 µL *volume* directly on top of the Pt working electrode and scanning between − 0.2 V and + 1.5 V at 100 mV/s for 3 cycles. The electrodes were rinsed using DI water and electrochemically cleaned before and after each test, and a baseline was created before each measurement.

In case of the vapor samples, each sample was taken from the top surface of the acetone liquid using a 50 mL syringe by filling the syringe with 25 mL acetone gas is manually delivered directly to the platinum surface above the electrode surface with a flow rate of 1 mL/s and scanning between − 0.2 V and + 1.5 V at 100 mV/s for 3 cycles. This was repeated for all prepared concentration and the electrodes were rinsed using DI water and electrochemically cleaned before and after each test, and a baseline was created before each measurement.

Moreover, we have used the closed glass system described in “Closed system for stability improvement” to test its effect on improving the stability of the readings. After Injecting 15 µL of 0.5 M H_2_SO_4_ on top of the electrode, the system is closed very tightly to prevent any leak and the vacuum pump is turned on to vacuum out any air until the pressure inside reaches stability. The baseline reading was taken by doing CV at 100 mV/s for 2 cycles. Acetone vapor with different concentrations were injected from the gas inlet with a flow rate of 2 mL/s, and CV was applied at 100 mV/s for 3 cycles after 2 min to make sure that the acetone vapor reaches the electrodes and ensure correct current readings. After each reading, the electrodes were cleaned using DI water and dried with nitrogen, and the system was cleaned by injecting nitrogen gas from the gas inlet for 2 min then vacuuming it from the other side, this was repeated 3 times to make sure that no acetone remains from the previous measurement.

## Results and discussion

### Sensor cyclic voltammetry results

**Figure 4:**
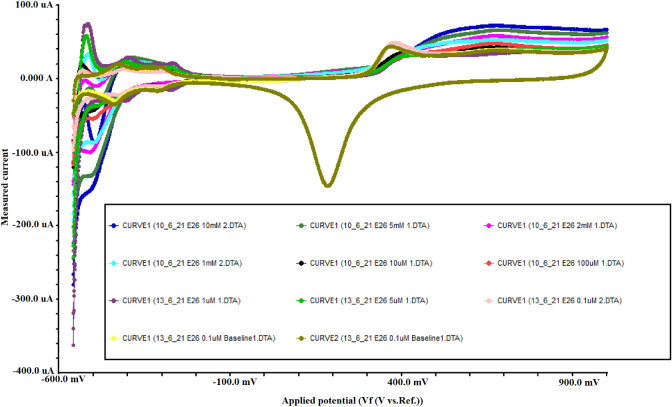
Cyclic voltammetry results for a range of (0–10 mM).

The cyclic voltammetry was done to measure the reduction current at each concentration, the results have shown the change in the reduction peak compared to the baseline after adding the acetone sample and the reduction current increases with the increase in acetone concentration. Figure 4 shows an example of the cyclic voltammetry results we collected for concentration range of (0–10 mM). The blank H_2_SO_4_ represent the baseline measurement (zero acetone level). The Acetone reduction current was measured at − 500 mV for most of the high concentrations range but shifted in an amount of ± 50 mV for the low concentrations. As shown in the cyclic voltammetry results, the scan starts at 0.2 V and sweeps negative and then positive to 1 V, the final sweep segment is then negative back to − 0.6 V. For all the measured concentrations the first wave of the butterfly is suppressed and the second wave is altered relative to the pristine hydrogen adsorption wave and the value of reduction current increases with the increase in acetone concentration.

### Sensor calibration results

**Figure 5:**
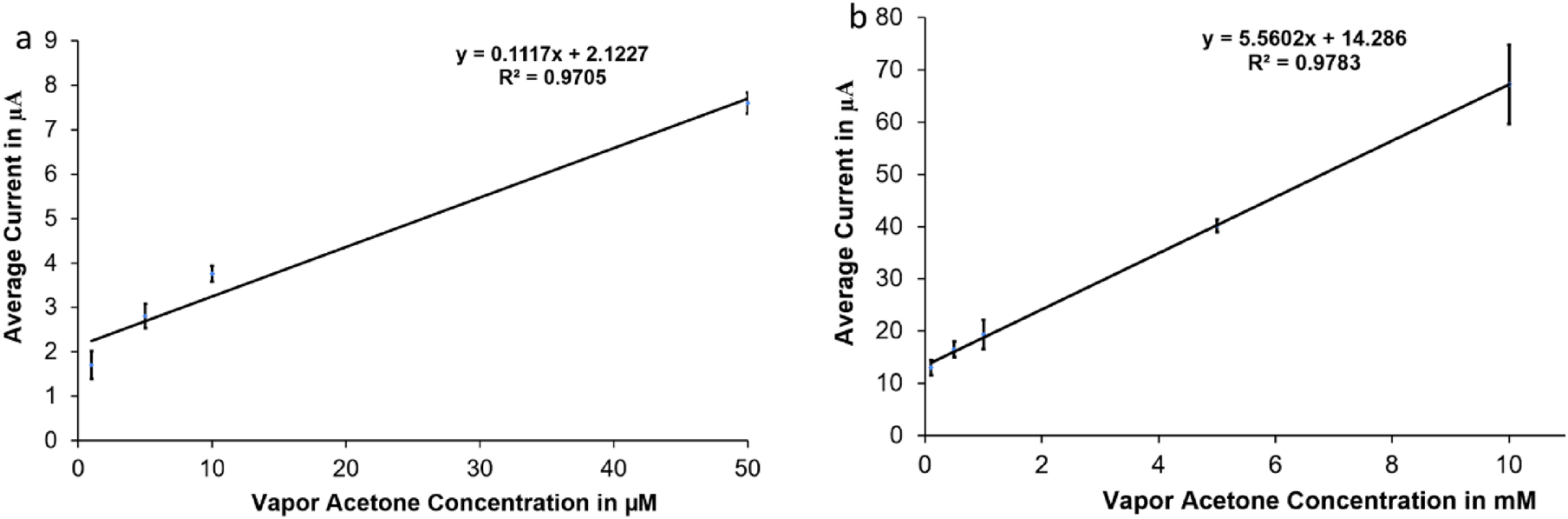
Calibration curves for vapor acetone: (**a**) small range (from 1–50 µM) (**b**) big range (0.5–10 mM).

The sensor calibration curves in Fig. 5 were obtained using the current values collected from the cyclic voltammetry results after subtracting the baseline collected from a blank H_2_SO_4_ in the butterfly region. We have created two calibration curves: the first one is for the small concentrations range (1–50 µM) with average currents in the range of (1.7 μA ± 0.31 μA) to 7.6 μA ± 0.24 μA) (Fig. 5a), the second one is for the high concentrations range of (0.5–10 mM) with average currents in the range of (13 μA ± 1.3 μA) to (67.23 μA ± 7.6 μA) (Fig. 5b). The sensor was able to measure in an acetone range of (1 μM–10 mM) with sensitivity of 0.1117 A/M and a correlation coefficient of 0.97 for the small concentration range, and a sensitivity of 5.56 μA/mM and a correlation coefficient of 0.978 for the high concentration range; the sensitivity was found by taking the slope of the calibration curve. The sensor output current increased with the increase in acetone concentration in a linear relationship in both low and high concentration ranges. The error bars in each calibration curve represent the standard deviation of ten calibration data points taken using ten different electrodes of same type under the same conditions. The variations of the measurement results might be caused due to the variations in the speed and direction of injecting acetone samples, the effect of fast acetone evaporation in the open air, the chemical binding of acetone on the electrode, and the electrochemical crosstalk between electrodes.

**Figure 6:**
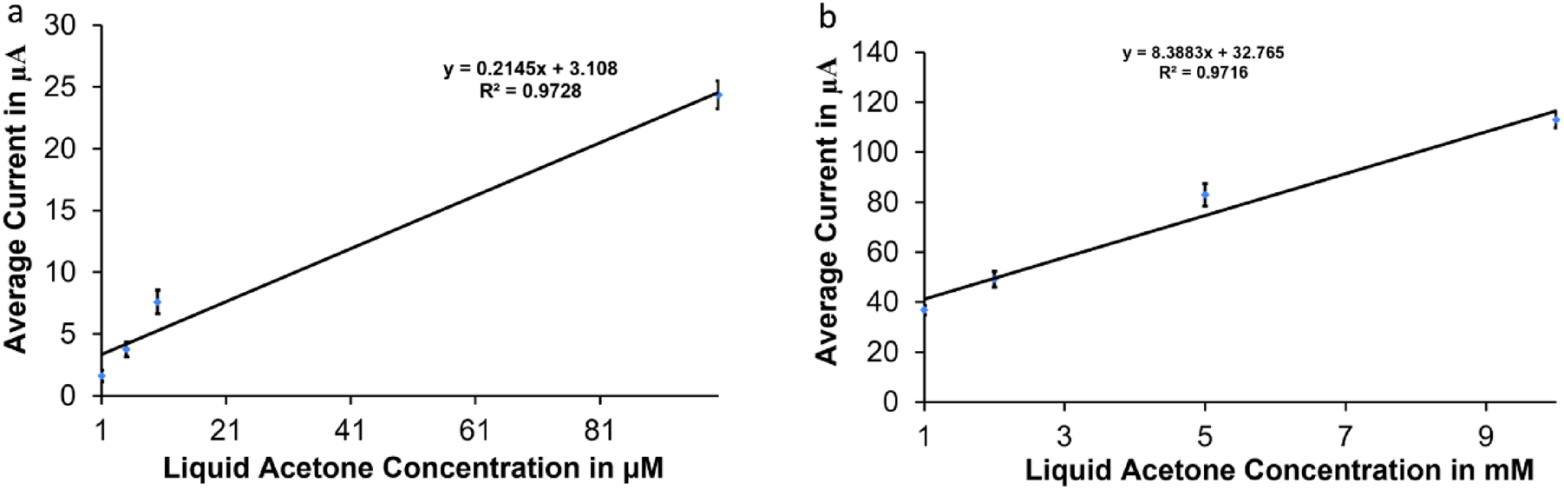
Calibration curves for liquid acetone: (**a**) small range (from 1 to 100 µM) (**b**) big range (1–10 mM).

The sensor calibration curves in Fig. 6 were obtained using the current values collected from the cyclic voltammetry results after subtracting the baseline collected from a blank H_2_SO_4_. We have created two calibration curves: the first one is for the small concentration range (1–100 µM) with average currents in the range of [(1.6 μA ± 0.47 μA) to (24.36 μA ± 1.14 μA)] (Fig. 6a), the second one is for the high concentration range of (1–10 mM) with average currents in the range of [(36.85 μA ± 1.85 μA) to (113 μA ± 3.2 μA)] (Fig. 6b). The sensor was able to measure acetone range of (1 μM to 10 mM) with sensitivity of 0.2145 A/M and a correlation coefficient of 0.973 for the small concentration range, and a sensitivity of 8.39 μA/mM and a correlation coefficient of 0.97 for the high concentration range; the sensitivity was found by taking the slope of the calibration curve. The sensor output current increases with the increase in the acetone concentration in a linear relationship in both low and high concentration ranges. The error bars in each calibration curve represent the standard deviation of ten calibration data points taken using ten different electrodes of same type under the same conditions. The variations of the measurement results might be caused due to the variations in the speed and direction of injecting acetone samples, the effect of fast acetone evaporation because the measurements were done in an open air, the chemical binding of acetone on the electrode, and the electrochemical crosstalk between electrodes.

### Sensor reproducibility

The calibration curves in “Sensor calibration results” demonstrate the responsiveness and reproducibility of the sensor signal to the change in acetone concentration in both vapor and liquid phases. The sensor has shown a good reproducibility when liquid samples were tested, with a mean of 113 μA and a standard deviation of 3.2 μA at the maximum acetone level of 10 mM, and a mean of 1.6 μA and a standard deviation of 0.47 μA at the low acetone level of 1 μM. Similarly, the sensor has shown a good reproducibility when vapor samples were tested, with a mean of 67.23 μA and a standard deviation of 7.6 μA at the maximum acetone level of 10 mM, and a mean of 1.7 μA and a standard deviation of 0.31 μA at the low acetone level of 1 μM.

### The results from the closed glass system

**Figure 7:**
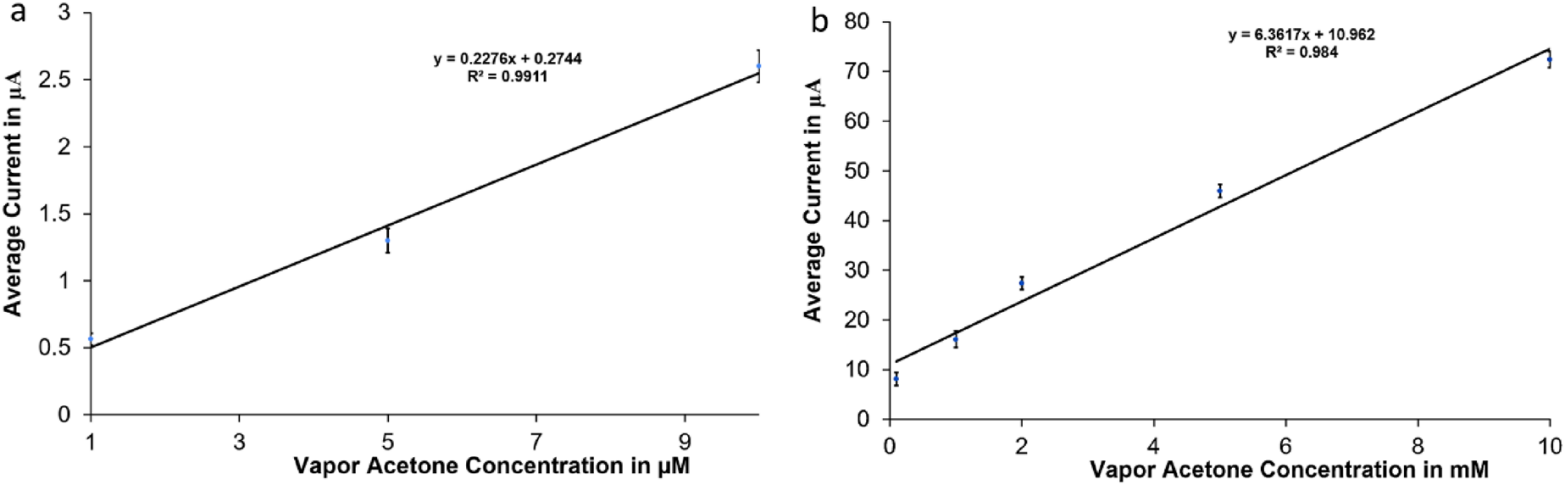
Calibration curves for vapor acetone: (**a**) small range (from 1 to 10 µM) (**b**) big range (0.1–10 mM).

The sensor calibration curves in Fig. 7 represent the results achieved from testing acetone vapor samples in the closed glass system. Figure 7a includes low concentration range (1–10 µM) with average currents in the range of [(0.56 μA ± 0.043 μA) to (2.6 μA ± 0.12 μA)], while Fig. 7b shows the high concentration range of (0.5–10 mM) with average currents in the range of [(8.1 μA ± 1.28 μA) to (72.4 μA ± 1.59 μA)]. The sensor was able to measure acetone range of (1 μM to 10 mM) with sensitivity of 0.2276 A/M and a correlation coefficient of 0.99 for the small concentration range, and a sensitivity of 6.4 μA/mM and a correlation coefficient of 0.98 for the high concentration range; the sensitivity was found by taking the slope of the calibration curve. The sensor output current increased with the increase in acetone concentration in a linear relationship in both low and high concentration ranges. The error bars in each calibration curve represent the standard deviation of six calibration data points taken using six different electrodes of same type under the same conditions. The variations of the measurement results might be caused due to the variations in the speed and direction of injecting acetone samples and the electrochemical crosstalk between electrodes. The results have shown a great improvement in the sensor stability, sensitivity, and linearity. The error in the readings could be reduced to about half of the values achieved for the measurements done in the open system, where the standard deviation is 0.23 and 3.2 μA at the acetone levels of 1 μM and 10 mM respectively. This indicate that this system was able to reduce the amount of the acetone exchange with the air and therefore it improves its stability.

## Conclusion and future work

In this paper, we have demonstrated the use of cyclic voltammetry to measure acetone concentration in liquid and vapor forms at disposable screen-printed electrodes. The sensor was shown to have a good linear response with respect to acetone concentration in both liquid and gas phases over a range of 1 µM to 10 mM. The calibration results achieved from liquid samples has a sensitivity of 214.48 μA/mM and a correlation coefficient of 0.973 for the small concentration range, and a sensitivity of 8.39 μA/mM and a correlation coefficient of 0.97 for the high concentration range. The calibration results achieved from the vapor samples has a sensitivity of 111.7 μA/mM and a correlation coefficient of 0.97 for the small concentration range (1–100 µM), and a sensitivity of 5.6 μA/mM and a correlation coefficient of 0.98 for the high concentration range (1–10 mM). The limit of detection for our acetone sensor is about 0.1 µM. The sensor has also shown a good reproducibility, with a mean of 113 μA and a standard deviation of (3.2 μA) at the 10 mM liquid acetone level and a mean of 1.6 μA with standard deviation of 0.47 μA at 1 μM vapor acetone level. Moreover, we have built a closed glass system to reduce the evaporation of acetone and the amount of error in the readings, the results have shown that our new system was successful in improving all the sensor features including stability, linearity, and sensitivity.

The results achieved in this paper are comparable to the results published by Wang et al.^1^ and Motsegood et al.^7^. The method used in Ref.^1^ is based on Amperometry at an applied potential of − 2.25 V by using a lead foil electrode in sodium tartrate solutions which is different from our method, and no details or images of the system were included. The results have shown a good linearity, but with lower sensitivity of 0.00207 in a range from 0.86 to 4.3 mM while we achieved a higher sensitivity in a wider range from 1 µM to 10 mM that includes the human physiological range. Moreover, they measured acetone in liquid phase only and their results show only one data point with no error bars indicating that they only did one experiment which is not enough for validating the operation of their system.

Our measurement method is very similar to what was published by Motsegood et al.^7^. But they didn’t include any details or images of the system they used and they measured acetone in gas phase only. Their results show good linearity but lower sensitivity and selectivity compared with our work. Also, their results include only one data point with no error bars indicating that they only did one experiment which is not enough for validating the operation of their system.

One disadvantage of the sensor system proposed in this paper is the need for H_2_SO_4_ to complete the reaction which makes its design less compatible with integration and multiplexed sensing applications, as a result this will make the design of a handheld device that includes this sensor harder especially for the applications that include diabetes monitoring. In future, we are going to design a complete system with an enzyme-based acetone sensor that is more compatible with integration for measuring breath acetone in the physiological range that could help in diabetes monitoring.
