# Author Correction: Acetone sensing in liquid and gas phases using cyclic voltammetry

**DOI:** 10.1038/s41598-023-28720-y

**Published:** 2023-01-31

**Authors:** Yusra Obeidat, Abdel Monem Rawashdeh, Ayman Hammoudeh, Rawan Al‑Assi, Ahmad Dagamseh, Qasem Qananwah

**Affiliations:** 1 Electronic Engineering Department, Hijjawi Faculty for Engineering Technology, Yarmouk University, Irbid, 21163 Jordan; 2 Department of Chemistry, Faculty of Sciences, Yarmouk University, P.O. Box 566, Irbid, Jordan; 3 Department of Biomedical Systems and Informatics Engineering, Hijjawi Faculty for Engineering Technology, Yarmouk University, Irbid, 21163 Jordan

Correction to: *Scientific Reports* 10.1038/s41598-022-15135-4, published online 30 June 2022.

The original version of this Article contained errors.

In the Materials and methods section, under the subheading ‘Sensor electrodes’,

“All experiments used the Palmsens screen-printed electrodes (Palmsens, Switzerland).”

now reads:

“All experiments used BVT screen printed electrodes (Palmsens, Netherlands).”

As a result, the legend of Figure 1 was incorrect.

“Palmsens screen printed electrodes.”

now reads:

“BVT screen printed electrodes.”

The original Article has been corrected.

